# 국내 COVID-19 입원 환자의 비만, 상승된 인터루킨-6와 입원기간의 상관관계

**DOI:** 10.7586/jkbns.23.025

**Published:** 2024-05-16

**Authors:** Hyunjung Oh, Kyoungsan Seo

**Affiliations:** 1 College of Nursing, Chungnam National University·Department of Nursing, Chungnam National University Hospital, Daejeon, Korea; 1 충남대학교 간호대학·충남대학교병원; 2 College of Nursing, Research Institute of Nursing Science, Chungnam National University, Daejeon, Korea; 2 충남대학교 간호대학·간호과학연구소

**Keywords:** COVID-19, Obesity, Interleukin-6, Length of stay, 코로나19, 비만, 인터루킨-6, 입원기간

## Abstract

**Purpose:**

The purpose of this study was to investigate the associations of obesity and clinical characteristics including interleukin-6 (IL-6) with hospital length of stay (LOS) in coronavirus disease-2019 (COVID-19) patients in Korea.

**Methods:**

A retrospective descriptive study design was employed to analyze medical data from a government-designated hospital in a city of Korea. Clinical data were collected from 256 patients with COVID-19 in negative-pressure isolation wards in 2021. The following parameters were analyzed: body mass index (BMI), IL-6 levels, age, sex, comorbidities, healthy habitsat the time ofadmission, and LOS. The statistical package SPSS 26.0 was used for descriptive statistics, the independent t-test, the chi-square test, and partial correlation coefficients.

**Results:**

The age of COVID-19 patients was positively correlated with BMI (r = -.16, *p* = .012), IL-6 levels (r = .14, *p* = .022) and LOS (r = .26, *p* < .001). Obesity, non-drinking, hypertension, and older age were associated with longer LOS.

**Conclusion:**

These results suggest that age, obesity, and hypertension in COVID-19 patients are related to LOS. Studies investigating other factors that can affect long-term hospitalization in COVID-19 patients are suggested.

## 서론

### 1. 연구의 필요성

코로나바이러스감염증-19 (coronavirus disease-2019, COVID-19)는 중증급성호흡기증후군 코로나바이러스 2 (severe acute respiratory syndrome coronavirus 2, SARS-CoV-2)에 의한 감염으로[1], 이 바이러스에 감염된 경우, 경증에서 중증까지 호흡기감염증상이 나타나며 특별한 치료 없이 회복되기도 하지만[2] 심각한 경우, 조절이 어려운 과도한 숙주 면역반응인 급성호흡곤란증후군(acute respiratory distress syndrome)을 일으켜 중증에 이르게 한다[3].

고령, 당뇨병, 고혈압, 암, 면역 저하, 비만, 심장질환, 폐질환 등의 유병은 COVID-19 감염의 위험성을 높이는 요인으로 지목되었다[4]. 특히 과체중 또는 비만한 COVID-19 감염 환자에서 병원 입원과 중환자 치료[5], 합병증 발병과 입원 기간 증가가 보고되기 시작하였다[6]. 최근 국외 연구에서 COVID-19로 입원한 환자에서 비만 유병률의 유의한 증가를 확인하거나, 증상이 있는 중증 환자 또는 응급 환자의 체질량지수(body mass index, BMI)가 비확진자보다 상대적으로 높은 것을 확인하였다[7,8]. 하지만 국내에서 BMI와 입원기간의 상관관계를 확인하는 연구는 거의 보고되지 못하고 있다.

비만이 COVID-19 감염증에 미치는 병리적 기전에 대해 다음과 같은 이론이 주요하게 적용되고 있다. 하나는 SARS-CoV-2 바이러스가 세포에 침투할 때 이용되는 효소인 안지오텐신 전환효소 2 (angiotensin-converting enzyme 2, ACE2)가 지방세포에 의해 생산되므로 비만한 사람에게서 ACE2가 상대적으로 많아 바이러스의 유입을 촉진 시킨다는 설명이다[9]. 또, 지방세포에서 많은 사이토카인(cytokine) 분비를 하므로 비만이 면역체계에 영향을 미쳐 감염을 유리하게 만들 수 있으며[10], 그 중 인터루킨-6 (interleukin-6, IL-6)는 급성기 염증에서 가장 중요한 사이토카인으로 염증성 바이오마커로서의 역할을 하며 특히 비만한 환자에서 중요한 중증 지표가 될 수 있다[11]. IL-6이 비만한 COVID-19 환자에서 높은 수치를 보이는 점이[12] 이전에 보고된 SARS-CoV, MERS-CoV 및 H1N1 influenza A 감염의 주요 특징으로 보고된 상승된 IL-6과 유사하다[13-15]. SARS-CoV와 유전적 유사성을 가진 SARS-CoV-2 바이러스[1]에 의한 감염도 비만한 환자에서 IL-6의 기능과 연관성이 있을 것으로 판단된다. 이에 비만한 COVID-19 환자에서 생리적 지표의 하나인 IL-6 수치를 함께 확인하는 것도 중요하다.

COVID-19 감염으로 중증 환자에 기여하는 위험 요인을 확인하면 실무에서 감염취약계층을 집중 관리할 수 있게 된다. 선행 연구에서 COVID-19 중증도에 기여하는 대상자 특성으로 75세 이상의 고령, 남성, 심한 비만, 암 등이 지목되었다[5,16]. 급성 폐 손상 환자의 비만이 입원기간 증가와 재활시설이나 요양시설로 퇴원에 기여하는 것으로 이미 보고되었다[17]. 최근 COVID-19 환자의 임상 특성을 파악한 국외 한 연구[18]에서 BMI 25kg/m^2^ 이상인 환자의 평균 입원기간은 약 14일로 BMI 25kg/m^2^ 미만 환자의 평균 9일과 비교하여 입원기간은 더 증가한 것으로 보고된 바 있다. 그러나 COVID-19 환자의 BMI 및 IL-6 사이의 관계, 입원기간 증가에 대한 영향을 탐색하는 연구들[8,19,20] 대부분이 평균 BMI가 높은 서양인을 대상으로 하였다. COVID-19의 영향력은 국가별 인종별로 다양한 스펙트럼을 보이고 있으므로 상대적으로 BMI와 비만 분류 기준의 차이가 있는 아시아인, 특히 한국인을 대상으로 COVID-19 환자의 비만과 입원기간에 대한 상관성을 파악하는 연구가 필요하나 국내 연구는 거의 없는 실정이다. 비만이 급성 감염성질환인 COVID-19의 발병과 임상 결과 중에 하나인 입원기간에 미치는 영향력을 탐색한 연구 결과는 국가 감염질환 관리와 임상 결과 지표 선정, 국민건강 정책의 주요한 기초자료가 될 수 있을 것이다.

### 2. 연구 목적

연구의 목적은 국내 COVID-19 진단 후 음압격리병상에 입원한 환자의 BMI, IL-6과 입원기간 간의 상관관계를 파악하고자 함이며, 구체적인 목적은 다음과 같다.

1) COVID-19 입원환자의 특성에 따른 BMI 및 IL-6, 입원기간 차이를 파악한다.

2) COVID-19 입원환자의 비만 정도에 따른 장기 입원 비율의 차이를 파악한다.

3) COVID-19 입원환자의 BMI 및 IL-6, 입원기간 간의 편상관관계를 파악한다.

## 연구 방법

### 1. 연구 설계

이 연구는 COVID-19 진단 후 음압격리병상에 입원한 환자의 전산화된 의무기록을 통해 BMI 및 IL-6, 입원기간 간의 상관관계를 파악하기 위한 후향적 조사연구이다.

### 2. 연구 대상

이 연구는 COVID-19 확진을 받은 국내 20세 이상 성인 대상자 중 국가에서 정한 기준에 따라 2021년 1월 1일부터 12월 31일 사이에 국내 일개 대학병원 국가지정 음압격리병상에 입실한 환자 전체를 대상으로 하였다. 그 중에 입원기간 2일 미만인 자와 19세 이하는 분석 대상에서 제외하였다. 해당 기간 동안 전자의무기록에서 COVID-19 진단 코드와 음압격리병상 수가 코드를 통해 분석 가능한 대상자는 270명이었으며, 분석하고자 하는 변수의 누락 값이 존재하여 자료가 불완전한 경우와 COVID-19 격리 해제 후 다른 사유로 장기 입원한 경우는 추가로 제외하고 최종 256명의 자료를 분석에 이용하였다. 연구대상자 수의 검정력을 G*power 프로그램을 이용하여 사후 분석하였다. 비만과 입원의 상관관계를 확인한 선행 연구[7,8]의 입원 발생률 28%를 근거로 중간 정도의 효과크기 0.25, 양측 검정, 유의수준 .05일 때 검정력은 .98로 나타났다.

### 3. 연구 도구

#### 1) 비만 (Obesity)

비만은 BMI로 측정 및 판단하며, 대한비만학회(Korean Society for the Study of Obesity)에서 제시한 계산식(체중/신장(m)의 제곱)에 따라 산출하였다[21]. 한국인의 비만 분류 기준에 따라 저체중군(< 18.5 kg/m^2^), 정상체중군(18.5-22.9 kg/m^2^), 과체중군(23.0-24.9 kg/m^2^), 1단계 비만군(25.0-29.9 kg/m^2^), 2단계 비만군(30.0-34.9 kg/m^2^), 고도비만군(≥ 35.0 kg/m^2^)으로 분류할 수 있다[21].

#### 2) IL-6

염증 사이토카인(pro-inflammatory cytokine)의 하나인 IL-6는 감염이나 조직손상 등에 대한 반응으로 체내에서 즉시 증가하며 방어기전을 작동시키는 물질로 면역반응에 직접 작용한다[22]. IL-6의 상승은 염증의 발생으로 해석할 수 있으며[22], 검사방법은 전기화학발광 면역측정법(electrochemiluminescence immunoassay)을 사용하였고 자료수집 기관의 참고치 기준인 7.0 pg/mL를 초과한 경우 IL-6의 상승으로 판단하였다. 여러 번 측정한 경우 입원 시 가장 초기에 측정한 수치를 선택하였다.

#### 3) 입원기간 (length of stay)

COVID-19 확진 환자가 국가지정 음압격리병상에 입실한 일자부터 격리 해제 또는 사망으로 음압격리병상에서 퇴실하는 날까지의 일수를 의미하며, 국가보건당국의 대응 지침에 근거하여[23] 대상자의 음압격리병상 입실과 퇴실이 결정되었다. 14일 이상 음압격리병상에 체류한 경우를 장기입원으로 분류하였다.

### 4. 자료 수집

자료수집을 위하여 국내 대전광역시 충남대학병원의 전자의무기록에서 연구자가 수기로 자료를 추출하였다. COVID-19 감염병이 폭발적으로 확산되고 치료제나 예방 백신 보급이 원활하지 않아, 국가 1급 감염병으로 지정되어 사회적 거리두기가 적용되던 2021년 전체 기간을 자료수집 기간으로 설정하였다. 2021년 일개 대학병원에 입원한 대상자 중 COVID-19 진단코드(D00033034, U071)와 음압격리병상 수가코드(AJ031, AK034, AK035)를 이용하여 대상자를 선별하였다. 의료정보이용신청서(연구검색용)를 자료수집 양식과 함께 기관 의무기록팀의 승인을 받고 부여받은 접근 권한 내에서 CDW (clinical data warehouse) 프로그램을 이용하여 자료를 수집하였다. 선행연구[5,16]를 바탕으로 성별, 연령(세), 신장(cm), 체중(kg), BMI (kg/m^2^), 흡연상태, 음주상태, 병력(당뇨병, 고혈압, 암 유무), 생화학적 검사지표(IL-6), 입원기간을 주요 변수로 구조화된 자료수집용 증례기록서를 가지고 수집하였다.

### 5. 자료 분석

연구자료의 분석은 SPSS 26.0 통계 분석 프로그램(IBM Corp., Armonk, NY, USA)을 이용하였고 유의수준 *p* < .05에서 유의성을 판단하였다. 대상자의 인구학적, 질병 특성과 BMI, IL-6, 입원기간은 서술통계를 이용하여 빈도, 백분율, 평균, 표준편차를 산출하였다. 대상자의 특성에 따른 BMI와 IL-6, 입원기간의 차이는 독립표본 t-test로 분석하였다. 대상자의 BMI에 따른 입원기간의 차이는 카이제곱 검정(chi-square test)으로 분석하였다. 대상자의 연령, BMI, IL-6, 입원기간의 편상관관계는 일반적 특성에서 영향을 줄 수 있는 변수들을 보정한 후 Pearson Correlation Coefficient로 분석하였다.

### 6. 윤리적 고려

자료수집은 자료수집기관의 연구윤리심의위원회의 승인(IRB File No. 2022-09-014)과 의료정보실에 자료 열람 권한 승인 요청 후에 진행하였다. 승인된 접근 권한 내에서 얻은 자료는 통계법 제33조(비밀의 보호)에 의거 명시된 사용 목적 이외는 사용하지 않았으며 연구대상자의 개인정보 보호를 위하여 연구 대상자 선별을 위한 의무기록번호(등록번호) 및 병리 번호는 별도의 파일로 보관하고 개인식별정보는 수집하지 않았다. 모든 자료는 코드화 처리하여 분석하였다.

## 연구 결과

### 1. 대상자의 특성

대상자의 성별은 남성 134명(52.3%), 여성 122명(47.7%)이었으며, 평균 연령은 56.01 ± 17.80세였다. BMI는 평균 24.89 ± 4.26 kg/m^2^로 저체중군 14명(5.5%), 정상체중군 75명(29.3%), 과체중군 44명(17.2%), 1단계 비만군 96명(37.5%), 2단계 비만군 18명(7.0%), 고도비만군 9명(3.5%)으로 분류되었다. 담배 흡연자는 47명(18.4%), 알코올 음주자는 85명(33.2%)이었다. 입원 시 기저질환으로 당뇨병 67명(26.2%), 고혈압 94명(36.7%), 암 18명(7.0%)이 있었다. IL-6 수준은 평균 58.22 ± 152.07 pg/mL이었으며, 비정상적으로 상승되어 있는 환자가 184명(71.9%)이었다. 총 음압격리병동 입원기간은 2일에서 37일로 평균 11.25 ± 6.72일이었고 14일 초과 입원 환자는 60명(23.44%)이 있었다(Table 1).

### 2. 대상자의 특성에 따른 BMI, IL-6, 음압격리병동 입원기간의 차이

대상자의 성별, 고혈압, 암 질환 유무에 따라 BMI가 통계적으로 유의한 차이를 보였다. 즉, 남성(t = 3.20, *p* = .002), 고혈압 진단군(t = 2.71, *p* = .007), 암이 없는 집단(t = -2.22, *p* = .027)의 BMI가 유의하게 높았다. IL-6은 연령, 당뇨병, 고혈압 유무에 따라 통계적으로 유의한 차이를 보였다. 즉, 65세 이상(t = -2.88, *p* = .005), 당뇨병(t = 2.54, *p* = .013), 고혈압(t = 2.02, *p* = .045)이 있는 경우 입원 시 IL-6 수치가 유의하게 기준치보다 상승되었다. 격리실 입원기간은 연령, 음주상태, 고혈압에서 통계적으로 유의한 차이를 보였다. 즉, 65세 이상(t = -5.04, *p* < .001), 비음주군(t = -2.72, *p* = .007), 고혈압 진단군(t = 2.56, *p* = .011)에서 입원기간이 더 길었다(Table 2).

### 3. 대상자의 비만 정도에 따른 음압격리병동 장기 입원의 차이

대상자의 비만 정도에 따라 장기 입원 유무 비율에 있어 차이가 있는지 확인한 결과 통계적으로 유의한 차이가 있었다(χ^2^ = 11.19, *p* = .048). 즉, 정상체중 집단에 비해 저체중과 과체중, 비만 1단계, 고도비만에서 장기 입원 환자의 비율이 높았다(Table 3). 블록 내 기대빈도가 5이하인 그룹을 제외하였을 때 정상 집단 대비 과체중과 비만 1단계의 대상자의 장기 입원 비율이 높게 나타났다(Table 3).

### 4. 대상자의 연령, BMI 및 IL-6, 입원기간 간의 편상관관계

대상자의 BMI, IL-6, 입원기간에 영향을 줄 수 있는 대상자의 성별, 고혈압, 당뇨병, 암, 음주습관을 보정하여 대상자의 연령, BMI 및 IL-6, 입원기간 간의 편상관관계 분석결과, 연령은 BMI (r = -.16, *p* = .012)와 통계적으로 유의한 부의 상관관계를, IL-6 수준(r = .14, *p* = .022)와 정적 상관관계를, 입원기간(r = .26, *p* < .001)과 통계적으로 유의한 정적 상관관계를 보였다(Table 4).

## 논의

이 연구는 일개 COVID-19 국가지정 음압격리병상에 1년간 입실한 환자의 의무기록에서 입실 시 나이, 비만 정도와 흡연 및 음주, 유병 질환과 IL-6 수치 등을 확인하고 이런 특성이 퇴실 시 임상 결과인 입원 기간의 장기화와 관련이 있는지를 분석하였다. 그 결과 연구 대상자의

BMI는 24.89 kg/m^2^이었고, 환자가 남성인 경우와 고혈압이 있는 경우 비만도가 높게 나타났다. 같은 COVID-19 대상자의 임상결과를 탐색한 선행 연구 중에 같은 아시아인인 중국의 연구[24]에 평균 BMI인 24.21 kg/m^2^와 유사하며, 영국에서 이루어진 연구[25]의 평균 BMI가 29.0 kg/m^2^보다 낮은 수준이다. 인구 집단 전체의 BMI 분포에 따라 COVID-19로 입원하는 환자의 BMI 분포가 달라진 것으로 보이며, 비만 정도가 낮은 아시안에서 비만이 입원 기간이나 환자의 임상 결과에 미칠 수 있는 영향력은 상대적으로 커질 수 있다. 또한 고혈압과 같은 만성질환이 있는 대상자에서 비만이 함께 있는 경우 COVID-19 감염병으로 음압격리병동에 입원할 가능성이 높아진 것을 확인하였고, 국가 감염관리정책에서도 고혈압이 있는 대상자의 중증화 위험을 고려할 수 있겠다.

한편, COVID-19 음압격리병상에 입실할 초기에 환자의 71.9%에서 IL-6의 증가가 관찰되었는데, COVID-19 감염 시 IL-6의 상승과 유의한 상관관계를 보고한 기존 연구들[12,26,27]과 일관된 결과를 보여준다. 이러한 결과는 SARS-CoV-2의 새로운 변이가 지속적으로 발생하고 있는 상황에서, IL-6의 지속적 모니터링이 환자의 임상 결과를 예측할 수 있는 바이오마커로 활용될 수 있음을 말한다[28].

연구에서 대상자의 평균 입원 기간은 11.25일로 나타났으며, 국외 선행 연구에서 보고된 평균 입원 기간인 8.6일보다 길었다[29]. 이러한 차이는 중증으로 진행되어 인공호흡기를 사용하게 되는 경우 입원이 장기화될 수 있는데, 이런 중증의 환자 발생은 사망률로도 이어질 수 있으므로, 입원기간 감시는 중증 환자 감시의 주요한 지표가 될 수 있겠다. 환자의 중증도나 장기 입원과 같은 임상 결과에 영향을 줄 수 있는 환자 개별 특성을 파악할 필요가 있겠다[30]. COVID-19 감염병 관리 정책에서 격리 해제기준[23]과도 입원 기간이 관련이 있을 수 있으나, 격리해제 후에도 바이러스가 지속적으로 검출이 되는 경우의 퇴실 기준 확보가 된다면 입원기간 단축에 기여할 수 있겠다.

음압격리병동 입원 기간은 정상체중 대비 과체중과 비만 1단계 집단에서 14일 이상 장기 입원 환자가 유의하게 많은 비율로 확인되었다. 다만, 연속 변수로 확인한 상관분석에서 입원기간은 대상자의 높은 연령만 관련이 있는 것으로 나타났다. 높은 BMI가 입원 기간의 증가에 유의미한 영향을 미치며, 이는 선행 연구에서 비만 환자들이 중증의 치료를 받을 가능성이 높고, 비만율이 증가할수록 중증도와 입원률, 사망률이 높일 수 있음을 확인한 연구 결과와 일치한다[31,32]. 한편, 저체중 집단의 경우도 높은 장기 입원 비율을 보였는데, 이것은 영양상태의 불량이나 다른 유병 질환을 가지고 있는 경우로 판단되며, 비만 환자뿐 아니라 저체중도 중증 환자로 이행의 주요한 위험 요인이 될 수 있으므로, 정상 체중 유지를 위한 포괄적 관리를 제안한다.

또한, 음압격리병동 입원 기간은 65세 이상에서, 비음주 집단과 고혈압이 있는 경우에 길게 나타났는데, 장기입원 중 60%가 여성 환자이었다. COVID-19의 중증도 및 사망률에 있어 성별 차이를 보고한 선행 연구도 있어[33], 추후 연구에서 성별에 따라 임상 결과에 영향을 줄 수 있는 요인을 따로 분석해보는 것도 실무에 도움이 될 수 있으므로 추가 연구로 제안할 수 있겠다. 여성에서 비음주자가 많았기에, 비음주 집단에서 입원 기간이 길어진 부분도 이와 관련이 있겠다. 한편, 고혈압 질환을 가진 경우, BMI가 높은 환자가 많고, 장기입원 환자가 많아지는 것을 확인하였으므로, 고혈압 질환자의 COVID-19 중증도를 예방하기 위해 체중관리를 제안할 수 있겠다. 이외에 평소 신체활동과 같은 건강생활습관 특성과 추가적인 인구사회학적 특성 중에 장기 입원에 영향을 줄 수 있는 요인을 파악하는 연구를 제안하며, 연구를 통해 수정 가능한 질병 중증도 영향 요인을 찾아내는 연구로 중증화 또는 입원을 예방하는 전략 마련을 할 수 있을 것이다.

대상자의 연령은 주요하게 입원기간 증가와 관련이 있는 요인으로 확인되었는데, BMI는 연령이 증가하면서 낮아지는 것으로 나타났기 때문에, 노령의 환자에서는 비만이 대상자의 중증도를 매개했다기 보다, 고령으로 인한 신체 상태가 장기 입원의 영향 요인으로 작용한 것으로 보인다. 이는 젊은 COVID-19 환자에서 비만이 좋지 않은 임상 결과에 영향을 준다는 연구와[34] SARS-CoV-2 바이러스의 높은 전염성은 사이토카인 폭풍으로 IL-6 상승과 노인의 경우 동반 질환이 있는 경우가 많았으며, 연령이 증가할수록 더 많은 치료와 중환자 치료가 적용됨에 따라 입원기간이 증가되었다고 한 연구[35]에서도 그 사례를 찾아볼 수 있다. 이러한 결과는 연령 및 동반 질환과 같은 환자 개인별 특성이 입원기간에 중요한 영향을 미치며, 이는 환자의 연령이나 동반질환, 비만과 같은 개인 특성별 맞춤 간호계획이 필요함을 시사한다[36-38]. 중증의 환자를 돌보는 임상 현장에서 환자의 감시와 적절한 치료와 신속한 중재를 제공하는 것은 중요하며, 그 근거로 마련할 수 있는 다양한 연구가 필요하다. 더불어, 중증의 환자가 되기 전에 위험 요인을 찾아 제거하고 관리하는 것 또한 COVID-19와 같은 대유행 감염병 관리에서 주요함을 다시 한번 확인할 수 있었다.

이 연구는 일개 국가지정격리병상의 입실 환자만을 대상으로 하여 분석하였기 때문에 연구 결과를 확대 적용하는데 주의를 기울일 필요가 있다. 1년 전체 기간을 그 대상으로 하여 계절이나 유행 정도가 미칠 수 있는 영향력은 최소화하였다. 후향적 방법으로 자료수집 되었기에 분석될 지표를 폭넓게 수집되지 못하였으므로 추후 연구에서 다양한 건강생활습관을 비롯한 환자의 특성을 추가하여 임상 결과를 파악하는 연구를 제안하며, 성별에 따라 장기입원에 영향을 미치는 요인들을 비교하는 후속 연구를 제안한다.

## 결론

이 연구는 국내에서 COVID-19가 대유행하여 국가가 그 질병을 통제하던 2021년에 국내 일개 COVID-19 국가지정격리병상에 입원한 환자의 의무기록을 후향적으로 분석하여 입실 전 지표인 동반 질환과 BMI, IL-6, 인구학적 정보, 흡연, 음주 정보 등을 기반으로 입원 기간이라는 임상 결과와의 관계를 후향적으로 탐색하였다. COVID-19 환자의 과체중과 비만은 장기 입원에 영향을 주며, 고혈압이 있는 경우나 남성에서 BMI가 높고, IL-6은 고령인 경우나 당뇨병이나 고혈압 질환이 있는 경우 높다. 나이와 입원기간은 정적인 상관관계가 있다. 고령, 성별, 비만, 만성질환이 COVID-19 환자의 입원 기간과 관련이 있으므로, 이를 치료 계획하고 제공하며, 중증 진행을 예방하는 데 활용할 것을 제안한다.

## Figures and Tables

**Table 1.** General Characteristics of COVID-19 Inpatients (N = 256)

Characteristics	Categories	n (%) or M ± SD	Min	Max
Sex	Male	134 (52.3)		
	Female	122 (47.7)		
Age (yr)		56.01 ± 17.80	20.00	95.00
	< 65	164 (64.1)		
	≥ 65	92 (35.9)		
Smoking	Yes	47 (18.4)		
	No	209 (81.6)		
Alcohol drinking	Yes	85 (33.2)		
	No	171 (66.8)		
Diabetes	Yes	67 (26.2)		
	No	189 (73.8)		
Hypertension	Yes	94 (36.7)		
	No	162 (63.3)		
Cancer	Yes	18 (7.0)		
	No	238 (93.0)		
Body mass index (kg/m^2^)		24.89 ± 4.26	15.16	37.44
Underweight	< 18.5	14 (5.5)		
Normal	18.5-22.9	75 (29.3)		
Overweight	23.0-24.9	44 (17.2)		
Obese class I	25.0-29.9	96 (37.5)		
Obese class II	30.0-34.9	18 (7.0)		
Severe obesity	≥ 35.0	9 (3.5)		
Interleukin-6 (pg/mL)		58.22 ± 152.07	1.50	1322.00
	≤ 7.0	72 (28.1)		
	> 7.0	184 (71.9)		
Length of stay (days)		11.25 ± 6.72	2.00	37.00
	≤ 14	196 (76.6)		
	> 14	60 (23.4)		

M = Mean; SD = Standard deviation; Min = Minimum; Max = Maximum.

**Table 2.** Body Mass Index, Interleukin-6, and Length of Stay according to Characteristics of COVID-19 Inpatients (N = 256)

Characteristics	Categories	Body mass index (kg/m^2^)			Interleukin-6 (pg/mL)			Length of stay (days)		
		M ± SD	t	*p*	M ± SD	t	*p*	M ± SD	t	*p*
Sex	Male	25.70 ± 3.76	3.20	.002	57.96 ± 118.61	-0.03	.977	11.56 ± 7.01	0.76	.446
	Female	24.01 ± 4.60			58.50 ± 182.41			10.92 ± 6.39		
Age (yr)	< 65	25.08 ± 4.24	0.93	.352	32.64 ± 66.55	-2.88	.005	9.59 ± 5.29	-5.04	< .001
	≥ 65	24.56 ± 4.30			103.80 ± 231.50			14.23 ± 7.90		
Smoking	Yes	25.22 ± 3.74	0.58	.560	78.40 ± 185.77	-1.01	.315	10.72 ± 7.43	-0.60	.550
	No	24.82 ± 4.37			53.68 ± 143.55			11.37 ± 6.57		
Alcohol drinking	Yes	25.37 ± 4.19	1.25	.212	56.13 ± 155.10	-0.16	.877	9.81 ± 5.26	-2.72	.007
	No	24.66 ± 4.28			59.26 ± 150.99			11.97 ± 7.25		
Diabetes	Yes	25.17 ± 4.26	0.61	.540	119.92 ± 266.10	2.54	.013	12.61 ± 7.42	1.94	.054
	No	24.80 ± 4.27			36.34 ± 68.35			10.77 ± 6.40		
Hypertension	Yes	25.83 ± 4.38	2.71	.007	86.44 ± 193.69	2.02	.045	12.77 ± 7.91	2.56	.011
	No	24.35 ± 4.11			41.84 ± 119.26			10.38 ± 5.76		
Cancer	Yes	22.76 ± 3.45	-2.22	.027	80.85 ± 121.04	0.65	.514	14.56 ± 10.30	1.44	.166
	No	25.06 ± 4.28			56.51 ± 154.24			11.00 ± 6.33		

M = Mean; SD = Standard deviation.

**Table 3.** Differences in Length of Stay according to Body Mass Index of COVID-19 Inpatients (N = 256)

Categories of obesity	Length of stay		χ^2^	*p*
	≤ 14 days (N = 196)	> 14 days (N = 60)		
	n (%)	n (%)		
Underweight	10 (71.4)	4 (28.6)	11.19	.048
Normal	64 (85.3)	11 (14.7)		
Overweight	32 (72.7)	12 (27.3)		
Obese class I	70 (72.9)	26 (27.1)		
Obese class II	16 (88.9)	2 (11.1)		
Severe obesity	4 (44.4)	5 (55.6)		

**Table 4.** Partial Correlation Coefficients between Body Mass Index, Interleukin-6, and Length of Stay (N = 256)

Variables	Age	Body mass index	Interleukin-6
	r (*p*)		
Body mass index (kg/m^2^)	-.16 (.012)	1	-
Interleukin-6 (pg/mL)	.14 (.022)	-.05 (.438)	1
Length of stay (days)	.26 (<.001)	.06 (.352)	-.01 (.863)

Control variables: sex, alcohol drinking, diabetes, hypertension, cancer.

## Data Availability

Please contact the corresponding author for data availability.
